# The association between effectiveness of the management processes and quality of health services from the perspective of the managers in the university hospitals of Ahvaz, Iran

**Published:** 2015

**Authors:** F Faraji-Khiavi, S Ghobadian, E Moradi-Joo

**Affiliations:** * Department of Health Services Administration, School of Health, Student Research Committee, Ahvaz Jundishapur University of Medical Sciences, Ahvaz, Iran

**Keywords:** knowledge management, quality of health services, hospital

## Abstract

**Background and Objective:** Knowledge management is introduced as a key element of quality improvement in organizations. There was no such research in university hospitals of Ahvaz. This study aimed to determine the association between the effectiveness of the processes of knowledge management and the health services quality from the managers’ view in the educational hospitals of Ahvaz city.

**Materials and Methods:** in this correlational and research, the research population consisted of 120 managers from hospitals in University of Medical Sciences Ahvaz. Due to the limited population, the census was run. Three questionnaires were used for data collection: Demographic characteristics, the effectiveness of knowledge management processes and the quality of medical services. To analyze the data, the Spearman association analysis, The Kruskal-Wallis, the Mann–Whitney U test, were used in SPSS.

**Results:** estimation of average scoring of the effectiveness of knowledge management processes and its components were relatively appropriate. Quality of medical services was estimated as relatively appropriate. Relationship of quality of health services with the effectiveness of knowledge management processes showed a medium and positive correlation (p < 0.001). Managers with different genders showed significant differences in knowledge development and transfer (P = 0.003).

**Conclusion:** a significant and positive association was observed between the effectiveness of knowledge management processes and health care quality. To improve the health care quality in university hospitals, managers should pay more attention to develop the cultures of innovation, encourage teamwork, and improve communication and creative thinking in the knowledge management context

## Introduction

Nowadays, various countries, organizations, and systems have started to join in the process of knowledge and they consider knowledge management as a method for improving the activities and performance of the company. On the other hand, their ability in production and application of knowledge are not open for public. Therefore, even the smallest industrial and service companies have a special attention to the concept of knowledge management and quality [**1**,**2**].

Rational considerations of the community have increased the demand for effective and high-quality services in the health system and consequently, improving the quality of services, accompanied by their cost, has been the most important goals for every hospital. For responding to a such updated requirement in the community and the health system, hospital information systems should move in the direction of cohesion and to supply the fundamental goals, namely to enhance the data quality, reducing the time of exchange, enhance in the satisfaction level and to increase the level of quality of service and, ultimately, to reduce the costs [**3**,**4**]. The quality is a type of policy that meets the needs of customers through the production of goods and services. This policy uses the organization’s resources effectively and efficiently and brings more profit for managers and staff in the Organization [**3**]. Several quality dimensions can be pointed out including the technical quality, the quality of the process (quality of a task or how to provide services), the quality of the infrastructure, the interactions quality, and the atmosphere quality and space [**5**].

On the other hand, knowledge management is considered as a unique component for economic growth of an organization [**6**]. Knowledge management is an important issue because it is concerned with the smallest valuable organizational capital, i.e., intellectual capital [**7**]. The absorption of knowledge elements and combining them with each other is an important management challenge, in a manner it flares up way spark of innovation and makes a new type of knowledge, i.e. the capital knowledge [**8**]. Gould considered the process of the knowledge acquisition, transfer, and application of management. In the field of knowledge management, these four provide teaching, feedback, and reeducation for an organization [**9**].

Studies showed that knowledge management has a considerable share in improving the quality of health services, increasing the efficiency and effectiveness of business actions, satisfaction of customer and improving decision-making; and the benefits of using knowledge management and improving the quality of health services made most organizations to do efforts for the implementation of these processes [**10**]. Managers and programmers of the regional health affairs can identify the health needs through the effectiveness of knowledge management and meet the medical needs of their covered population [**11**].

AJUMS is a major university in Iran and some of its hospitals are regional pole for health care in the southwest Iran. Hence, improving services’ quality is the main goal of the university. 

In some management literatures, the knowledge management has been introduced as one of the main pillars of improving quality in organizations. It seems that the quality of services can be improved through the implementation of effective knowledge management. This research after estimating the mean score for components of knowledge management in hospitals, introduced the components which were correlated to service quality. Since no research was conducted in this field in the health sector, so, this research aimed to specify the association among the effectiveness of knowledge administration processes and the health quality from the view of the managers of university hospitals in Ahvaz. 

## Materials and Methods

This correlational research performed during 2015. Study population included 130 managers of 5 educational hospitals in the AJUMS. Due to the limitation of statistical society, the whole population was studied using census methods. Respondents included directors and managers of hospitals, matrons, supervisors, the managers of all wards, and also supervisors of the accounting, social work, radiology, nutrition, service quality, pharmacy, laboratory, the personnel affairs, and reception units 

Data collection tools included three questionnaires: socio-demographic characteristics, effectiveness of knowledge management processes and quality of medical services. The validity of data collection tools was confirmed by 5 opinion leaders and experts. Socio-demographic characteristics questionnaire included age, sex, education, and work experience. The questionnaire of the effectiveness of knowledge management processes contained six components: tendency towards development and knowledge transfer, continual learning in the organization, perception of the organization as an overall system, people-oriented approach, the development of an innovative culture and development of competence and merit-based management [**12**]. This questionnaire contained 23 questions. Cronbach’s alpha for the questionnaire was 0.084. Care quality questionnaire devised by Asadi et al has 5 dimensions of technical quality, process quality (task quality or how to provide services), quality of infrastructure, quality of interactions, quality of the atmosphere and space [**5**]. This questionnaire consisted of 29 questions. Cronbach’s alpha was calculated at 0.90% to questionnaire of health care service quality. Scoring of questionnaire of knowledge management effectiveness and quality of service were estimated based on the Likert scale. In results, mean scores between 4 and 5 interpreted as appropriate; between 3 and 4 as relatively appropriate; between 2 and 3 as relatively inappropriate; between 1 and 2 as inappropriate.

Data were not normally distributed. For data analysis, Spearman correlation, Kruskal-Wallis and Mannwhitney investigations employed in SPSS. In addition, descriptive statistics and indicators of the mean and standard deviation were used to show the results. 

## Results

Out of 130 people, 120 people completed the questionnaire and the answer rate was 92.31. Respondents’ socio-demographic features are presented in **Table 1**.

**Table 1:** Socio-demographic profile of the respondents

1 Socio-Demographic variables	2 Categories	3 Number	4 Percentage
5 Age (years)	6 25-35	7 63	8 52.5
	9 36-45	10 48	11 40
	12 >45	13 9	14 7.5
15 Gender	16 Male	17 36	18 30
	19 Female	20 84	21 70
22 Marital status	23 Single	24 24	25 20
	26 Married	27 96	28 80
29 Academic degree	30 MSc.	31 26	32 21.6
	33 BSc.	34 80	35 66.7
	36 Associate degree and Diploma	37 14	38 11.7
39 Work experience	40 <10	41 53	42 44.2
	43 11-20	44 52	45 43.3
	46 >20	47 15	48 12.5

**Fig. 1:**
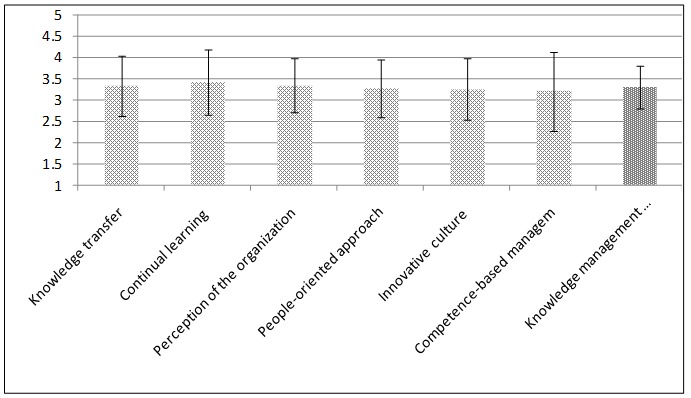
Average scores of the effectiveness components of knowledge management processes

**Table 1** shows that most respondents were under 35 years of age and had a work experience less than 10 years. Most of them were MScs. Most respondents were female and married. **Fig. 1** depicts the average rates of the effectiveness of knowledge management and its components.

**Fig. 2:**
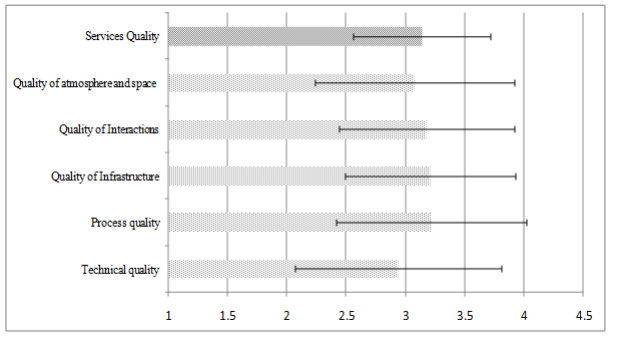
Average scores of therapeutic service quality components

**Fig. 1** depicts that the average rates of all the components of effectiveness of knowledge management were between 3 and 4, and estimated relatively appropriate. Continuous learning and competency development had the highest and lowest scores, respectively. However, effectiveness of knowledge management estimated relatively appropriate. **Fig. 2** displays the average scoring from the perspective of health managers is.

**Fig. 2** shows that the organizational-technical quality with a score less than three has been estimated by managers to be relatively inappropriate, but other dimensions of quality and whole service quality with a score more than 3 has been estimated as relatively appropriate. Table 2 displays the correlation of the effectiveness of knowledge management processes with quality of service

**Table 2:** Correlation of the effectiveness of knowledge management processes with health service quality

knowledge management effectiveness	Statistical indicators			Quality of Services			
		Technical	Process	Infrastructure	Interactions	Atmosphere and space	Total
Knowledge transfer	Spearman corr.	-0.018	0.126	0.132	0.044	0.079	0.104
	P-value	0.848	0.171	0.150	0.634	0.388	0.256
Continuous learning	Spearman corr.	0.184*	0.256**	0.157	0.165	0.124	0.213*
	P-value	0.044	0.005	0.087	0.072	0.179	0.019
perception Of the Organizational	Spearman corr.	0.282**	0.259**	0.348**	0.234*	0.393**	0.386**
	P-value	0.002	0.004	0.001<	0.010	0.001<	0.001<
Innovative culture	Spearman corr.	0.374**	0.257**	0.254**	0.104	0.252**	0.305**
	P-value	0.001<	0.005	0.005	0.258	0.006	0.001<
People-oriented approach	Spearman corr.	0.206*	0.139	0.187*	0.148	0.255**	0.236**
	P-value	0.024	0.131	0.041	0.106	0.005	0.01
Competency –based management	Spearman corr.	0.111	0.206*	0.182*	0.199*	0.190*	0.231*
	P-value	0.226	0.024	0.047	0.029	0.038	0.011
Total	Spearman corr.	0.246**	0.269**	0.293**	0.205*	0.332**	0.347**
	P-value	0.007	0.003	0.001<	0.025	0.001<	0.001<
*P < 0.05							
**P < 0.01							

**Table 2** shows that there was a medium and positive association between the quality of healthcare with the effectiveness of knowledge management processes (p < 0.001). Some of the components of knowledge management, components such as continuous organizational learning, organizational perception, development of innovative culture, people-based approach, the development of competency, were in medium size and positive association with health service quality. The highest association was between the components of the organizational perception with the atmosphere and space quality dimension (P < 0.001). The effectiveness of knowledge administration showed a direct and medium association with the atmosphere and space and showed a small association with other dimensions of the quality of health services (P ≤ 0.025).

In terms of the estimation of the effectiveness of knowledge management processes and quality of health care, people with different demographic characteristics did not show significant differences. However, the estimation of male and female respondents of the development and transfer of knowledge showed a significant difference (P = 0.003). Mean scores of men and women were 3.66 ± 66 and 3.42 ± 0.71, respectively. 

## Discussion

The effectiveness of knowledge management processes and all of its components were estimated relatively appropriate in hospitals of Ahvaz. In a study on the public relations’ school, Tsai found a direct and main association among knowledge adminstration procedures and systematic efficacy [**13**]. In mentioned study knowledge adminstration suggested as a significant parameter to predict the systematic efficacy; furthermore, knowledge application and transfer components were announced as the strongest predictors in knowledge management processes for organizational effectiveness [**13**]. In studies conducted on 301 different organizations in China, Zheng et al. have expressed that the design of a knowledge management project usually involves organizational change and organizations that accept adaptability with values, being involved with the staff, and joint missions in their culture, have a large desire to resolve the issues, find new ways of reducing costs, look to the future and to perform actively in their methods [**14**]. 

Knowledge management implementation of in various systematics initially needs organizational identification interrelated parameters that have the specific and unique characteristics to achieve systematic efficacy; on the same reason, the needed information and knowledge should be accessed by managers and staff in a different time [**15**]. While hospitals are considered more as service organizations, educational hospitals must concentrate on knowledge management in order to approach their educational as well as service providing goals, effectively. In addition, due to the specialized services it seems that even non-educational hospitals should use processes of knowledge management more effectively to show greater organizational effectiveness. In this health subsection, it is recommended that hospitals assemble teams composed of managers, professionals, and employees to transfer knowledge through organization.

From the view of managers, treatment quality in Ahvaz hospitals was relatively appropriate. Studies in health centers and hospitals in Yazd and Zanjan cities from the perspective of patients showed health services quality were estimated relatively appropriate [**16**,**17**].

Hospital managers can create mechanisms in order to increase the quality, improve the performance and satisfy patients and other stakeholders by providing and implementing interdepartmental projects for sharing information and technology and information systems with members. Quality is developing by measures that are determined by the processes of globalization and advances in information technology [**18**]. Thus, producing adequate data on the satisfied of the client’s understanding of service feature can assist systems to recognize the size that influences the system’s aggressive interest, and to stop the loss of support. After conducting studies in health centers in Ireland, Dotchin and Oakland estimated all components of quality, based on the perception of respondents, at appropriate level [**19**]. Hospitals are providing much more complex and wide services than health centers; thus providing a good quality in hospitals is much more challenging. In this study, technical dimension was relatively inappropriate, so, hospitals should focus on hospital’s structure indicators including training, and competence of staff, the technical condition of buildings and equipment, proper planning, develop new ways to assess customer needs organization to improve the technical quality [**20**].

In this study, the effectiveness of knowledge management processes showed significant association with health care quality. Among the components of knowledge management, development of innovative culture and organizational perception showed the highest correlation with quality of care. Innovative organizational culture includes innovation, encouraging teamwork and communication and organizational perception includes the creative thinking, the interactive and open dialogue; so, to modify the health care quality, proper measures needs to be taken to develop mentioned components by hospital managers [**21**]. 

Choi and Lee observed that most people in the public organizations of China are not willing to transfer knowledge to others and this can interfere with the process of knowledge management [**22**]. It seems that managers may be followed as a role model when they transferring their knowledge to colleagues and staff. This can be effective in order to transfer the tacit individual knowledge and organizational obvious knowledge. In addition, through planning, regulation, and policy-making, they can create a framework for easy access to information and knowledge, and increase speed of acquisition of knowledge in their organizations to achieve a better quality of care. To improve health care quality, hospitals must create a constructive structure for organization and provide a possibility for enjoying from its achievements to all organization's stakeholders [**15**,**23**]. Effectiveness of knowledge management processes showed the highest correlation with the elements of the atmosphere and space. Since spending long hours in medical environments is usually a stressful experience for staff, patients and visitors, designing green spaces around hospitals creates a happier environment by reducing pollution, improving physical and mental health of the patient; on the one hand, care of the patient can be easier for the hospital staff, and patient recovery time and costs can be reduced [**24**].

Since the majority of the managers of the study population had a university degree, they may have a better understanding of the effectiveness of knowledge processes and use it in order to develop a culture of innovation, organizational understanding, and creativity to achieve the desired quality of health care. Some related instructions, workshops, and educational materials for these managers may facilitate creating mentioned culture. 

## Conclusion

Significant, positive, and medium association was seen between the effectiveness of knowledge management processes with quality health care. It is suggested that the managers pay attention to the development of a culture of innovation, encouragement of teamwork, improvement of communication, creative thinking, and mutual dialogue within the framework of knowledge management to modify the health quality services in the university hospitals.

**Acknowledgement**

This article is the result of a research project approved 93s113 number of Ahvaz University of Medical Sciences Research Center and the research was carried out with the financial support and technology deputy of the university. Thereby we would like to thank the respected authorities and managers of different levels of Ahvaz University of Medical Sciences hospitals who cooperated in this study. 
